# Brokering the core and the periphery: Creative success and collaboration networks in the film industry

**DOI:** 10.1371/journal.pone.0229436

**Published:** 2020-02-27

**Authors:** Sándor Juhász, Gergő Tóth, Balázs Lengyel

**Affiliations:** 1 Agglomeration and Social Networks Lendület Research Group, Centre for Economic and Regional Studies, Hungarian Academy of Sciences, Budapest, Hungary; 2 Institute of Economics and Economic Development, Faculty of Economics and Business Administration, University of Szeged, Szeged, Hungary; 3 Spatial Dynamics Lab, University College Dublin, Dublin, Ireland; 4 International Business School, Budapest, Hungary; Universitat de Barcelona, SPAIN

## Abstract

In collaboration-based creative industries, such as film production, creators in the network core enjoy prestige and legitimacy that are key for creative success. However, core creators are challenged to maintain diverse access to new ideas or alternative views that often emerge from the network periphery. In this paper, we demonstrate that creators in the network core can increase the probability of their creative success by brokering peripheral collaborators to the core. The argument is tested on a dynamic collaboration network of movie creators constructed from a unique dataset of Hungarian feature films for the 1990–2009 period. We propose a new way to capture brokers’ role in core/periphery networks and provide evidence that being in the core and at the same time bridging between the core and the periphery of the network significantly increases the likelihood of award winning.

## Introduction

Creative work takes place in teams, where collaboration greatly impacts the breadth and depth of how ideas and skills can be combined [1, 2]. Therefore, a vast literature of network analytic research has looked at the structure of relationships in which individuals and teams are embedded to understand how network positions influence success [3–9].

A highly reflected concept to explain success in cultural fields and creative industries is the core/periphery structure of social relationships [4, 10]. A core/periphery network is characterized by a densely connected, highly central, cohesive subgroup of core actors and a set of peripheral actors that are only loosely connected to each other and to the core [11, 12]. It is often argued that individuals located in the selective core enjoy the social capital concentrating on a relatively small number of established players with the necessary material resources, political influence and social connections to enforce their central role in the creation of cultural and creative products [13, 14]. Core network position correlates with a prestige that signals legitimacy, experience and credibility [15–17] and can influence how artistic or creative work is received by the audience. The periphery, on the other hand, consists of a wider variety of actors who are less constrained by social relations and thus are more likely to develop alternative views or non-canonical concepts [10]. Ideas originating in the high-prestige core spreads faster and more easily throughout the community [18], whereas ideas coming from the lower prestige peripheral creators need more time for recognition and must filter through many more intermediaries [16, 17].

Consequently, being in the core rather than being in the periphery of the collaboration network provides different advantages for creative achievement. Previous empirical research has indeed confirmed that intermediate positions between the core and the periphery provide the highest chance of success [4, 19, 20]. Arguably, these special positions are beneficial, since individuals can combine the advantages of both parts of the network. Being close to the core might help the recognition of creative work. At the same time, being at least few steps away from the core can help individuals escape high social pressure to conform or find new creative opportunities [21, 22].

In order to succeed, individuals at the periphery must follow different strategies to collaborate on new projects than creators in the core. Peripheral creators need to become more visible and introduce their image and voice [4, 23]. Consequently, they are motivated to collaborate with core individuals to legitimise their ideas and work in the community. On the contrary, the challenge for established core creators is to maintain the novelty of creative production [24, 25].

In this paper, we demonstrate that core creators in the creative industries can gain an increased likelihood of achieving creative success in case they manage to bridge peripheral creators to the core. We argue that brokering between the core and the periphery of the network can bring the benefits of both worlds to core creators.

The advantages of brokers in collaboration networks are widely recognized and stem from the intermediary positions of individuals between loosely connected communities, which provide access to a diverse set of new ideas and opportunities [26]. From a strategic behaviour point of view, two distinct orientations describe how brokers can mobilize social capital. The structural hole theory emphasizes the *tertius gaudens* orientation [27, 28]. It suggests that the unique ties of brokers allow for mediation and control of the flow of ideas, information, knowledge and resources in the network that brokers can use for their own benefit [26, 29, 30]. In contrast, the *tertius iungens* orientation emphasizes the role of brokers in linking previously unconnected actors or in facilitating new examples of collaboration between connected individuals [29]. This latter theory has been used to explain how brokerage foster creativity, innovation, and success in a variety of contexts [31–33].

Based on the previous core/periphery and brokerage literatures, we propose that individuals who establish links between the core and the periphery gain more access to novelty than core individuals who do not have such connections. The latter group is likely to be too entrenched in the prevailing conventions of the community of important creators and thus tends to ignore the potential contributions of new ideas and knowledge from outside [4, 19, 34]. Consequently, core creators that bridge the core and the periphery with a *tertius iungens* attitude can be expected to have a high likelihood of achieving creative success for two reasons: Firstly, they have access to high prestige groups that can help them propagate their creative products. Secondly, their openness to working with peripheral creators can foster their creativity and innovation.

*Hypothesis*: *Core creators are more likely to achieve creative success in case they are network brokers such that they link the core to the periphery.*

To test this hypothesis, we construct time-varying collaboration networks of Hungarian movie creators from 1990 to 2009 and explain how core/periphery and brokerage positions influence the likelihood of winning awards in the major festival for new movies in Hungary. To define the creators’ position in the core/periphery continuum, we choose to apply the algorithm by Borgatti and Everett [11], while brokerage is captured through the edge-betweenness based measure of Everett and Valente [35]. Moreover, we construct a new Gatekeeping index that measures how much creators act as bridges between core and peripheral nodes in their ego network. By a regression framework with three-way interactions we search for the combined influence of core/periphery position with Brokerage and Gatekeeping. We find evidence that core creators with ties that bridge the entire network, and at the same time connect core and peripheral nodes, enjoy additional likelihood of creative success.

## Datasets, key variables and methods

### Data

The context of our empirical exercise is provided by the Hungarian film industry, which has improved its international reputation recently by winning two American Academy Awards in successive years and has collected various other prestigious honours in Europe including *Golden Palm (Palm D’or)* and the *Golden Bear*.

Counting awards is a well-established means of measuring individual creative performance in the tradition of creativity research [36, 37]. We collect data from the Hungarian film yearbooks (http://mandarchiv.hu/cikk/4643/Filmevkonyv) and operationalize creative success through the awards made at the *Hungarian Film Week*, the most prominent film festival in the country up until the recent past. The *Hungarian Film Week* was established in 1965 and (with a short hiatus during the 1970s) was a central event in the national film production for decades. By the 1990s, the festival’s programme had become like many other international film festivals and besides the main prize, directors, cinematographers, editors and writers were awarded for the quality of their performance by a select jury of film critics, producers and industry peers. Due to a drastic change in the financial support system for Hungarian movie production, the industry totally stopped in years 2012 and 2013. Our dependent variable is *Award-winning*, which equals 1 in a given year if the movie creator won an award in an individual category (e.g. best cinematography or best editing) and zero otherwise. In cases when the movie won the best movie prize, every creator gets a dummy of 1 in our dataset.

**Fig 1:**
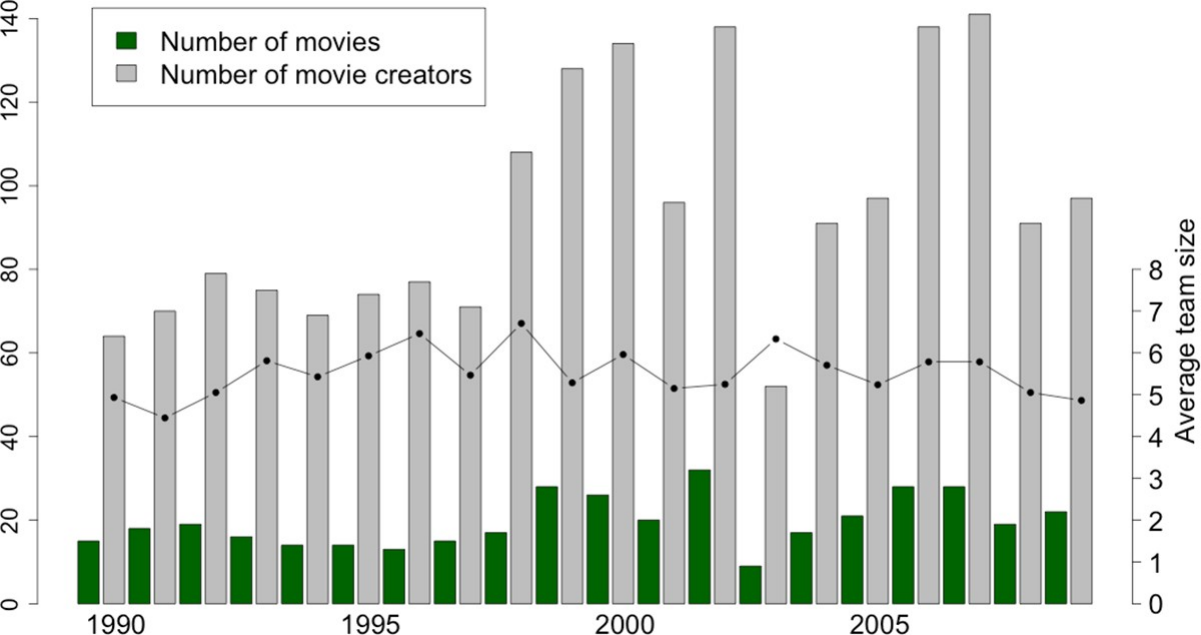
Collaboration patterns in movie production. We consider movie creators as the cinematographers, directors, editors, producers and writers of films. Histograms refer to the number of movies and creators; the dots linked by solid line refers to average team size in each of the years of the period under investigation.

To construct the bipartite collaboration network of movie creators, we collect data from the online *Hungarian Film Archive* (http://mandarchiv.hu/tart/jatekfilm). Since movie creation is project-based, the film industry’s collaboration networks are constantly created and re-created as individuals collaborate on a specific project, disband when the project ends, and then combine for a new project, often with new partners [38]. We analyse the unipartite projection of the bipartite affiliation network of movie creators and movies, where a link between any two movie creators indicates collaboration on a movie. In a similar fashion to Cattani and Ferriani [4], we considered movie creators as the following members of the production crew: cinematographer, director, editor, producer and writer. Fig 1 shows that the number of movies and the number of movie creators that produce a movie in the given year were fluctuating during the 1990–2009 period; however, the average team size of movies considering these key roles was relatively stable.

**Fig 2:**
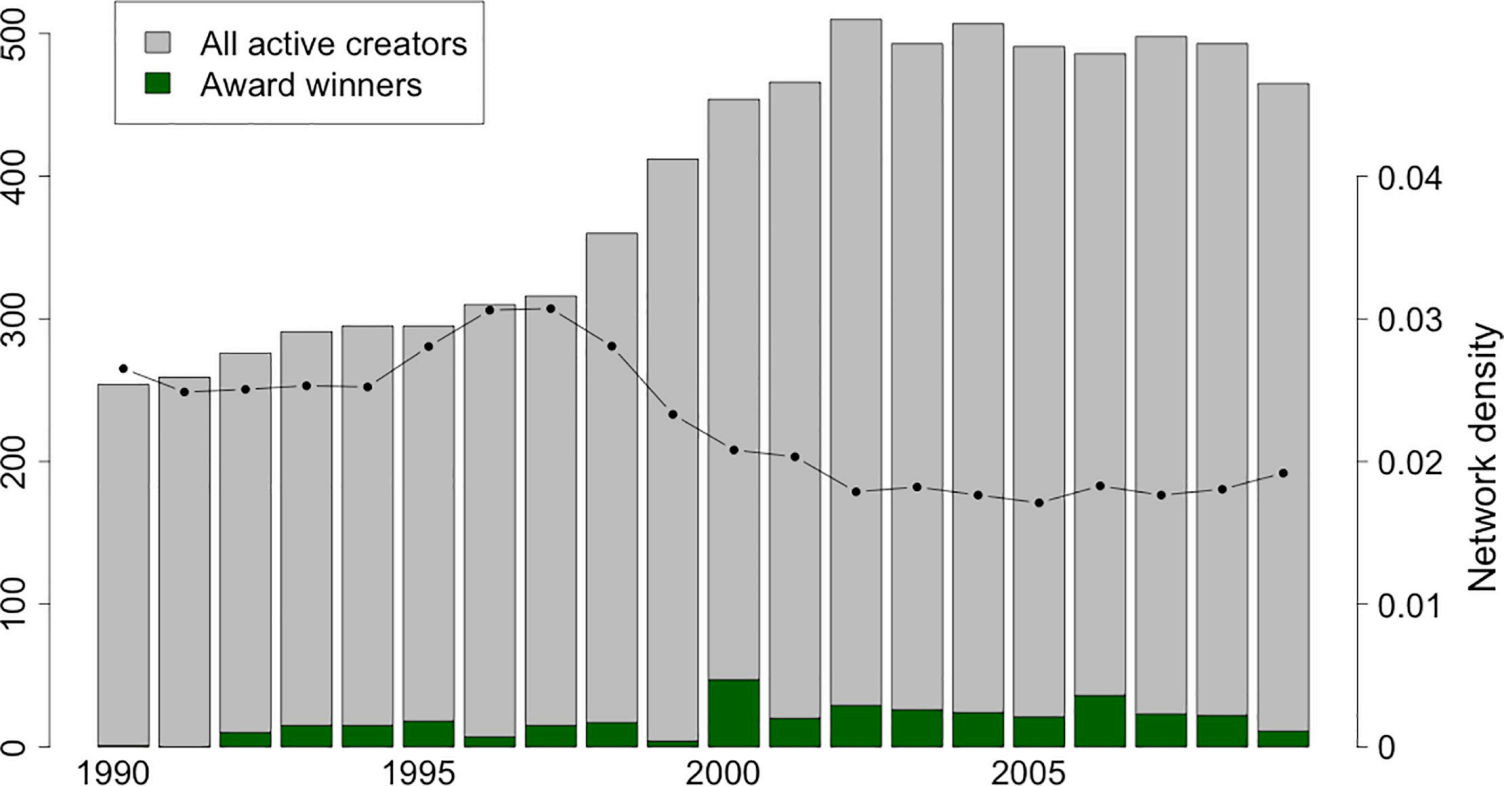
Active creators, award winners and network density. The numbers of active movie creators are based on 7-year moving-windows and represent our final sample. Award winners are also part of the active creator group. Histograms refer to the number of creators; the dots linked by solid line refers to average team size.

To create the collaboration network, we assume that the relationships formed through the production of a movie last for 7 years, similarly to other network studies related to creative production in movies or musicals [e.g. 4, 7]. Thus, a link is present in the adjacency matrix of the collaboration networks in the given year if the two movie creators have been worked together over the 7-year interval preceding the year in focus. For example, the collaboration network in 1990 covers cooperation in the 1984–1990 period. Fig 2 presents the number of active movie creators per year based on 7-year moving windows. As the number of active individual movie creators nearly doubled from early 1990s to early 2000s, the density of the collaboration network decreased over the years. The share of award winner individuals in all the active creators per year are presented in Fig 2 as well. On average more than seventeen creators won an award each year. Our sample contains 361 award winners out of 7672 individual movie creators along the period of 1990–2009. For more precise estimations we excluded the year without award winners from our analysis.

### Variables

We measure all individual creators’ positions on a core/periphery continuum using Borgatti and Everett’s [11] algorithm. Following the approach in previous work on movie collaboration networks of Cattani and Ferriani [4] and on individual innovation by Dahlander and Frederiksen [19], we estimate each node’s degree of coreness by the continuous measure of core/periphery. Accordingly, our *Coreness* indicator refers to the degree of closeness to a densely connected network core for each movie creator. We apply the described procedure to all network matrices of years 1990–2009. For the sake of straightforward interpretation we standardized *Coreness* into *z*-scores.

To quantify *Brokerage*, we use the betweenness-based measure recently suggested by Everett and Valente [35]. We opt to apply this indicator in the main analysis as it defines brokerage by considering how individuals connect otherwise loosely connected parts of the entire network, not only those in their immediate neighbourhood. *Brokerage* is computed in two steps. First, we calculate the edge betweenness centrality measure for every tie in the network. Second, for each node we assign a *Brokerage* score that is the average of the edge centralities which are incident to it. The indicator takes high value if the focal actor has ties that are part of many shortest paths in the whole network. For the sake of variable comparison, we standardized *Brokerage* into a *z*-score. As a robustness check, we repeat the exercise with *Brokerage* defined by Burt’s network constraint indicator [27] (see S4 Table).

**Fig 3:**
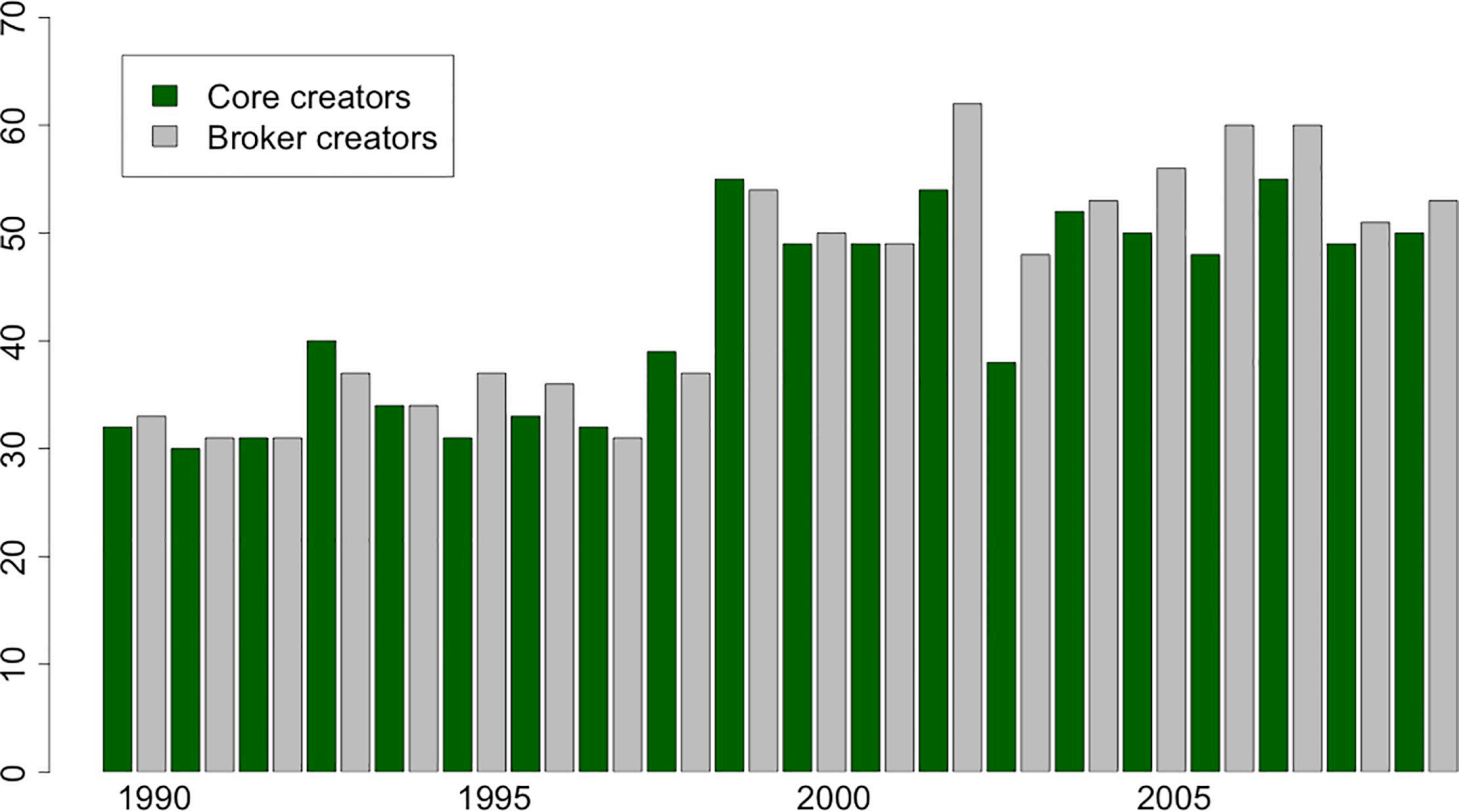
Change in the number of core and broker creators. The numbers of movie creators are based on 7-year moving-windows. The numbers of core and broker creators are based on the *Core* and *Broker* dummy variables.

For a more detailed understanding on how core/periphery position and brokerage jointly influence actors’ individual success, we created dummy variables from the *Coreness* and *Brokerage* indices in a similar fashion to Cattani and Ferriani [4]. The dummy variable is called *Core* that takes the value 1 in case the continuous *Coreness* value of the individual is in the top ten percentile of the measure’s scale (above 0.90) and zero otherwise. The *Broker* dummy which takes the value of 1 in the case of *Brokerage* is in the top ten percentile of the measure’s scale (above 0.90). These binary variables enable more precise estimation of interaction effects in regression frameworks than continuous variables. Fig 3 presents the number of *Core* and *Broker* creators along the examined period. Based on the applied dummy variables, the number of Core and Broker movie creators are nearly identical in every year.

**Fig 4:**
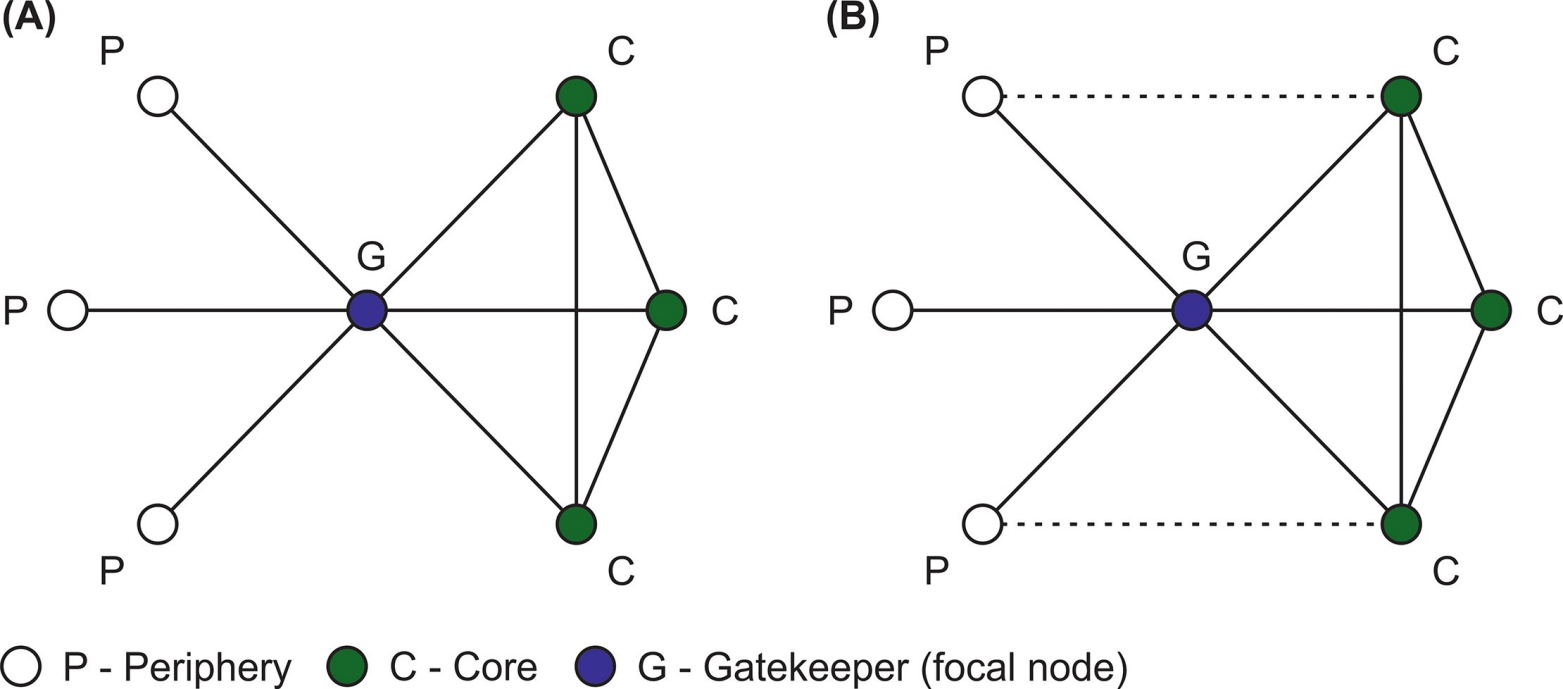
Illustration of high (A) and low (B) *Gatekeeping* indicators. G–stands for Gatekeeper creator, C–stands for Core creator and P–stands for Peripheral creator. (A) represents a possible situation in which the focal node acts more like a Gatekeeper (Gatekeeping = 0.9), while in case of (B) the focal node is less of a Gatekeeper (Gatekeeping = 0.7).

As edge betweenness based measure of *Brokerage* does not, of itself, help the identification of brokers between core and periphery at the global network scale, we also develop a new measure to further look at bridging positions between core and periphery in the ego network of creators. Therefore, we also introduce a new measure that we call *Gatekeeping*. The indicator refers to the extent to which the focal nodes’ ties are the only connections between core and peripheral nodes in its ego network. Eq (1) summarises the construction of the *Gatekeeping* measure.

$$Gatekeeping_{i}=1-\frac{L_{cp}+1}{\|v_{c}\|\times \|v_{p}\|+1}$$ (1),

where *L*_*cp*_ refers to the observed number of links between core and peripheral actors in the ego network of *i*, without the focal actor. In the denominator, ‖*v_c_*‖ refers to the number of core individuals in the ego network of creator *i* and ‖*v_p_*‖ is the number of peripheral nodes in the ego network of creator *i*. The indicator is the inverse of the observed ties between core and peripheral actors compared to the number of possible ties between the two types of neighbours. Fig 4 illustrates two hypothetical cases. In case of Fig 4(A), the focal nodes’ gatekeeping indicator has a relatively high value (*Gatekeeping* = 0.9) as there are both core and peripheral nodes in its’ ego network, but the focal node is the only connection between them. In case of Fig 4(B), the focal nodes’ gatekeeping indicator has a lower value (*Gatekeeping* = 0.7) as some of the peripheral neighbours are directly connected to core neighbours of the ego. In case of Fig 4(B), one can expect less benefit from connecting the core with periphery than in the case of Fig 4(A) because core neighbours can enjoy additional advantages residing at peripheral collaborators through their direct connections.

The *Gatekeeper* indicator identifies those movie creators who connect the core and periphery in their ego networks. This is a dummy variable that takes value 1 in case the value of Gatekeeping indicator is in the top ten percentile of the measure’s scale (above 0.90) and 0 otherwise. This binary variable enables us to test interaction effects in a precise way, which is a key step in our analytical strategy.

Additionally, we use several further variables as controls. Because of the applied 7-year moving-windows to create more stable and connected creator networks, we control for the number of *Films Per Window* on which the given network structure is based on. Moreover, we use the variable *Creators Per Window* in order to control for the number of active movie creators in the 7-year period or the number of nodes in the network in the given year.

Professionals new to the industry might receive disproportional attention from award voters, who may prefer new talents to veteran creators [4]. To account for this effect, a *Newcomer* dummy variable was created for each movie creator in every year which takes 1 if a professional is a new participant of movie production and 0 otherwise. Length of careers can also determine the bankability of movie creators [39]. Therefore, we create the *Experience* variable to provide a control for the years spent in the industry since the creators’ first movie. Since success breeds success, individual creators are more attractive for colleagues if they have worked with prestigious collaborators or they have already won an award [40, 41]. Therefore, we construct the variable *Previous award* that provides a control for the number of awards the creator has won before.

Finally, we applied year fixed effects and individual role fixed effects as well, which refer to the main roles of the creators in the period being classified as cinematographer, director, editor, producer or writer categories. Detailed descriptive statistics of our variables, their correlation matrix and distribution of the *Gatekeeping index* can be found in the supplementary information (S1 Table, S1 Fig and S2 Fig). While *Coreness* is neither correlated to *Brokerage* nor to *Gatekeeping*, *Brokerage* and *Gatekeeping* are correlated. Therefore, we do not focus on the influence of *Gatekeeping* alone, but rather its combined effect with *Coreness* and *Brokerage*.

The combination of *Brokerage* and *Gatekeeping* is rather necessary because none of these indices can capture the role and benefits of brokers in core/periphery networks on their own. On the one hand, *Brokerage* in itself is unable to quantify bridging between core and periphery. The indicator cannot distinguish whether core creators link core with periphery or bridge two or more loosely connected core groups. On the other hand, *Gatekeeping* is myopic by definition and only considers bridging inside the ego network of creators and does not consider the nodes’ position in the global network. Two core creators with identical *Gatekeeping* values can have different access to novel ideas from the periphery indirectly through their connections. This limitation of the *Gatekeeping* indicator is illustrated in S3 Fig. In case a node has high value of *Brokerage* and high value of *Gatekeeping*, it means that its ties are the only connections between core and periphery in the ego network, while they are also important shortest paths to bridge different parts of the global network.

### Estimation strategies

Our dependent variable *y_i_* takes the value of 0 when creator *i* in year *t* did not win an award and 1 if creator *i* won an award in year *t*. We estimate a pooled logistic regression model with year fixed effects and a fixed effect on the role of creators in movies and cluster standard errors at the creator level. The regression model is defined by:

$$Pr(y_{it}=1)=\alpha +\beta_{1}{Coreness}_{it}+\beta_{2}{{Coreness}^{2}}_{it}+\beta_{3}{Brokerage}_{it}+\beta_{4}{Gatekeeping}_{it}+\beta_{5}Z_{it}+\varphi_{t}+\omega_{i}+u_{it}$$ (2),

where *Coreness* (and its squared term to test the non-linear effect), *Brokerage*, and *Gatekeeping* denote the network characteristics of creator *i* at year *t*. *Z_it_* is for the collection of control variables. *φ_t_* is a year fixed effect and *ω_i_* is a categorical fixed effect for the main role of creators’ in movie production (e.g. director or cinematographer).

In the second part of the analysis, logistic regressions are run with dichotomized *Core*, *Broker*, and *Gatekeeper* variables and their dyadic and three-way interactions to capture the creators’ role as a broker from different perspectives in a core/periphery setting:

$$Pr(y_{it}=1)=\alpha +\beta_{1}{Core}_{it}+\beta_{2}{Broker}_{it}+\beta_{3}Gatekeeper_{it}+\beta_{4}({Core}_{it}\times {Broker}_{it})+\beta_{5}({Core}_{it}\times Gatekeeper_{it})+\beta_{6}({Broker}_{it}\times Gatekeeper_{it})+\beta_{7}({Core}_{it}\times {Broker}_{it}\times Gatekeeper_{it})+\beta_{8}Z_{it}+\varphi_{t}+\omega_{i}+u_{it}$$ (3)

where besides the introduced dichotomized variables we use the same model setting.

Applying binary variables is an appropriate approach for introducing variable interactions for three reasons. First, the interaction of more than two continuous variables makes interpretation difficult and requires similar variable distributions. Second, significant estimates for binary interactions yield significant and even higher point estimates for continuous measures. Third, binary variables are less correlated with each other than are continuous variables. It is important to highlight that the application of a three-way interaction is necessary to verify our hypothesis on whether core and broker creators are more likely to achieve creative success in cases where they bridge the core and the periphery of the network.

## Results

**Fig 5:**
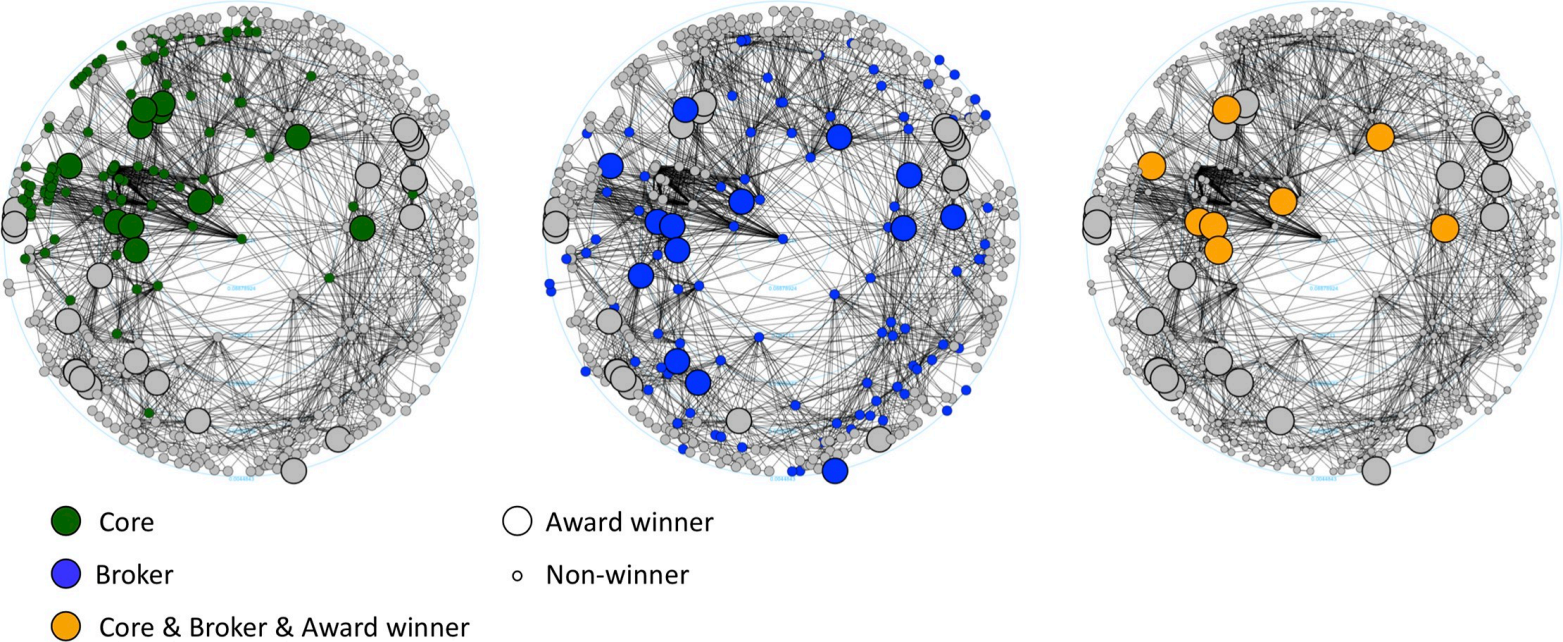
Representation of core, broker and award winner creators in the network of 2006. Uniformly large nodes with a wider outline represent award winners in all three graphs. Colours of the nodes show their special positions as Core, Broker and Core & Broker & Award winner creators (see Legend). Nodes with a higher degree of centrality are closer to the center of the circular layout. The network is based on a 7-year moving window, where nodes represent movie creators and edges represent collaboration on all movies made during the 7-year period. The layout is created by the *graphlayouts* R package.

Fig 5 represents the creator network of 2006 as an example. One can see that non-core and non-broker creators also won awards, but individual creators are more likely to win, if they are both in the core and are brokers at the same time. However, our main question is whether core creators are more likely to win in cases where they broker peripheral creators to the core.

Table 1 presents the absolute numbers and the share of award winners in the different creator groups underpinning our intuitions detailed above. Around 5% (361 from 7672) of creators won an award over the period analysed. The share of award winner creators is notably higher (16%) in cases in which they manage to combine the benefits of core and broker positions by comparison with the ‘only core’ (7%) and ‘only broker’ (4%) individuals. An even higher 20% share of creators (57 out of 349 individuals) won an award in cases where they are in the core, broker and gatekeeper positions at the same time.

**Table 1:** Share of award winner creators by core, broker, and gatekeeper positions.

	Award winner (n = 361)	No winners (n = 7311)	Full sample (n = 7672)	Share of award winners (5%)
**Non-core, Non-broker & Non-gatekeeper**	155	5253	5408	3%
**Only core**	84	1104	1188	7%
**Only broker**	10	251	261	4%
**Only gatekeeper**	1	42	43	2%
**Core & Broker**	64	330	394	16%
**Core & Broker & Gatekeeper**	57	292	349	20%

The numbers of movie creators are based on 7-year moving-windows. The number of core, broker and gatekeeper creators are based on the *Core*, *Broker* and *Gatekeeper* dummy variables. As there was no winner in 1991, we excluded the year from our analysis.

Table 2 presents the coefficients of four logit regression models described in Eq 2. Control variables are included in all models and we introduce explanatory variables in a stepwise manner. In model (1) we introduced both linear and quadratic forms of *Coreness* into the estimation as it was suggested by Cattani and Ferriani [4]. The coefficient of *Coreness* is significant and positive, while the quadratic term correlates negatively with the dependent variable. This finding means that creators are more likely to receive an award for their contribution to a movie in any given year in cases where they are closer to the core of the network; however, proximity to the core has diminishing returns.

**Table 2:** Network position and individual success–results of logit regressions.

	Dependent variable–award winning				
	(1)	(2)	(3)	(4)	(5)
**Coreness**	0.859***		0.876***		0.568***
**(z-score)**	(0.088)		(0.081)		(0.088)
**Coreness**^**2**^	-0.092***		-0.092***		-0.030**
**(z-score)**	(0.021)		(0.017)		(0.013)
**Brokerage**		0.402***	0.413***		0.148**
**(z-score)**		(0.054)	(0.047)		(0.073)
**Gatekeeping**				0.884***	0.695***
**(z-score)**				(0.065)	(0.086)
**Creators Per Window**	0.017**	0.012	0.017*	0.019**	0.023**
	(0.009)	(0.009)	(0.009)	(0.009)	(0.009)
**Films Per Window**	0.022	0.044	0.028	0.020	0.006
	(0.057)	(0.057)	(0.057)	(0.058)	(0.058)
**Newcomer (dummy)**	2.321***	2.280***	2.504***	2.763***	2.849***
	(0.144)	(0.138)	(0.145)	(0.153)	(0.147)
**Experience**	0.047***	0.032***	0.012	-0.005	-0.023*
	(0.009)	(0.010)	(0.011)	(0.011)	(0.012)
**Previous awards**	0.532**	0.518*	0.545**	0.404	0.477**
	(0.256)	(0.292)	(0.241)	(0.255)	(0.223)
**Constant**	-15.923***	-16.576***	-16.350*	-16.208***	-16.024***
	(5092)	(5.123)	(5.094)	(5.214)	(5.233)
**Year FE**	Yes	Yes	Yes	Yes	Yes
**Role FE**	Yes	Yes	Yes	Yes	Yes
**BIC**	2686.907	2752.501	2627.678	2608.370	2543.442
**Log likelihood**	-1218.219	-1255.488	-1184.132	-1183.423	-1137.541
**N**	7672	7672	7672	7672	7672

Standard errors in parentheses * p<0.10, ** p<0.05, *** p<0.01

Standard Errors are clustered at the creator level.

We introduce *Brokerage* in model (2) without controlling for *Coreness*. The positive and significant coefficient indicates that *Brokerage* significantly induces the creators’ likelihood of award winning. Most importantly, neither the significance level nor the direction of correlation changes for either the *Coreness* or *Brokerage* variables in model (3). Results reported in model (3) suggest that creators are more likely to receive an award when they are closer to the core and their broker status further increases the probability of award winning.

In model (4) we test the importance of our *Gatekeeping* variable on award winning, independently from *Coreness* and *Brokerage*, while in model (5) we include *Gatekeeping* in our extended model. Already in line with our *Hypothesis*, which will be further examined in Table 3, the coefficients in the final model version show similar results to the previous models and *Gatekeeping* also shows significant positive effects. These results indicate that *Coreness* together with both *Brokerage* on the global network level and *Gatekeeping* in the ego network influence creative success in core/periphery networks. Consequently, these findings support the idea that being part of the network core and brokering the core and the periphery provide complementary benefits for creative workers.

**Table 3:** Relationship between core and broker position and award winning.

	Dependent variable–award winning						
	(6)	(7)	(8)	(9)	(10)	(11)	(12)
**Core**	1.638***			1.509***	1.298***		1.145***
	(0.124)			(0.137)	(0.149)		(0.143)
**Broker**		1.641***		1.397***		1.046***	0.744**
		(0.182)		(0.295)		(0.317)	(0.349)
**Gatekeeper**			2.051***		1.841***	1.935***	0.594
			(0.152)		(0.362)	(0.202)	(1.036)
**Core X Broker**				2.699***			2.378***
				(0.198)			(0.426)
**Core X Gatekeeper**					2.556***		2.184***
					(0.166)		(0.213)
**Broker X Gatekeeper**						2.264 ***	1.926***
						(0.187)	(0.340)
**Core X Broker X Gatekeeper**							2.509***
							(0.182)
**Constant**	-48.028***	-46.205***	-49.736***	-51.424***	-52.232***	-50.543***	-13.721***
	(17.035)	(16.758)	(17.006)	(17.003)	(17.127)	(16.958)	(1.437)
**Controls**	Yes	Yes	Yes	Yes	Yes	Yes	Yes
**Year FE**	Yes	Yes	Yes	Yes	Yes	Yes	Yes
**Role FE**	Yes	Yes	Yes	Yes	Yes	Yes	Yes
**BIC**	2710.52	2755.46	2683.06	2635.49	2624.50	2686.85	2697.33
**Log lik.**	-1228.37	-1265.91	-1229.71	-1196.98	-1191.49	-1222.66	-1276.38
**Observations**	7672	7672	7672	7672	7672	7672	7672

*Source*: Author’s own construction.

Standard errors in parentheses * p<0.10, ** p<0.05, *** p<0.01 Further control variables that are not reported in the table include Creators per Window, Films per Window, Newcomer, Experience and Previous awards. Standard Errors are clustered at the creator level.

The Bayesian information criterion (BIC) improves by the inclusion of our main explanatory variables indicating model improvement. The variance inflation factor (VIF) test indicates no serious problems of multicollinearity (S2 Table in the supplementary information).

To test our *Hypothesis* more accurately, we investigate the coefficient of the interaction terms between the three dichotomized main indicators in Table 3. We introduce our dummy variables stepwise in model (6), model (7) and model (8) and test the interactions of *Core*, *Broker* and *Gatekeeper* variables in a stepwise manner again from model (9) to model (12).

We find that the dichotomized indicators have the expected positive significant coefficients in models (6), (7), and (8). The dyadic interaction terms in models (9), (10), and (11) are found to further increase the probability of award winning, which confirms our previous findings in Table 1. Finally, the strong, positive and significant coefficient of the three-way interaction effect in model (12) indicates that creators who are part of the Core and are also Brokers at the same time have an especially high likelihood of award winning in cases when they act as Gatekeepers between the core and the periphery. These findings demonstrate that core creators can have the highest chance of achieving creative success if their ties are on many of the shortest paths in the global network and if the same ties represent the few connections between core and peripheral neighbours in their ego network. This result verifies our *Hypothesis* and suggests that being in the core of a community and bridging the core to the periphery significantly helps creative success.

## Robustness checks

To validate our results, we applied a variety of robustness checks. We tested the k-core indicator of nodes, as an alternative measure for network coreness (S3 Table). Similarly, we tested constraint as an alternative measure for network brokerage (S4 Table). Both alternative indicators provided us with very similar results confirming major findings reported in the main text.

Network indicators are usually highly correlated. *Degree* often captures the co-variance of other node-level measures, and our case is no exception (see S1 Fig). Therefore, we also provide models in which the degree of nodes is included, especially since many of our variables correlate with it. S4 Table shows our final models with and without *Degree*. *Degree* takes the significance of *Brokerage* in case we use the edge betweenness measure, but not in those where the constraint-based *Brokerage* measure is applied. The coefficient of the *Coreness* indicators does not change when *Degree* is included in the model. Similarly, *Gatekeeping* is still significant in both models, which confirms our main argument that brokering between the core and the periphery increases the chances of creative success.

Since we are focusing on the success of individuals rather than teams, we also tested our models by removing best picture winners and keeping award winners only in individual categories (such as best cinematographer or editor). In this exercise, we only have 127 award winners over sixteen years. Applying the same 7-year moving-window setting, our results still hold in the stepwise settings. Although indicators loose significance in the final model, our new Gatekeeping variable has a positive and significant coefficient verifying our argument (see S5 Table in the supplementary information). In a further step, we tested a variety of randomized settings with random awards, randomly rewired networks or totally random networks and all these test results, taken together, strengthened the robustness of our findings (see S6 Table for details). Furthermore, we got similar results when applying different estimation strategies such as rare event logit regression models (S7 Table) or individual level fixed effect models (S8 Table).

Aggregating data over time enables us to consider the persistence of professional relationships; nonetheless, varying moving-windows can greatly influence our results [42]. Therefore, we tested our main models with 5-year and 9-year moving windows and found similar results (see S4 Fig and S9 Table). Finally, we test the Core and Broker dummies with cut off points at 0.75 percentiles, by which our main results did not change significantly (S10 Table).

## Discussion

In this paper, we aim for a novel understanding of how core creators can take advantage of brokerage in core/periphery networks. Our empirical exercise is based on a unique and open access dataset of Hungarian feature films, from which we created time-varying collaboration networks of individual movie creators. By applying a novel approach to consider brokerage between core and periphery, our results confirm that creators have the highest chance to achieve creative success if they belong to the network core and bridge the core to the periphery at the same time. This core and broker position in the network support creative success by providing complementary benefits for central creators.

The study contributes to the network-based research on creativity, innovation and success. It addresses the highly researched core/periphery trade-off [4, 10, 19] from the point of view of core individuals and presents an alternative explanation as to how they can maintain their creative edge through bridging ties. Our findings suggest that core individuals with *tertius iungens* orientation to bring together people from different parts of the network could help them to foster creativity, innovation, and thereby increasing the chances of their own success [31–33]. Moreover, the study makes a small contribution to the emerging field of ‘science of success’ [43] by combining different network indicators to determine creative success.

As a methodological improvement, we provide a simple measure we call *Gatekeeping* to help the identification of brokers between the core and the periphery of the network. Certainly, this ego network-based measure can be further developed, and additional methodological research is needed to generalize the mechanisms of brokerage in core/periphery structures. Our analytical approach is based on the three-way interaction effects of *Coreness*, edge betweenness based *Brokerage* and *Gatekeeping* to map their combined influence on creative success. This enabled us to measure how ties of core creators that bridge different parts of the network and also represent connections between core and peripheral individuals contribute to their success. However, the development of a single variable to capture bridging between core and peripheral nodes considering the global network structure would be an elegant and useful contribution for future research.

The findings of this study are based on a specific era of the Hungarian film production that is certainly not in the core of the global feature film industry. Even though the observed community covers a relatively isolated industry, the international success of Hungarian artists or co-working relationships in foreign movie productions has provided access to external social capital for the Hungarian filmmakers, which is invisible in our data. A more puzzling question is whether the same results would hold if one looked at the global movie production, in which there are probably more than one core group of artists with their own peripheries [44]. Brokerage between core groups might offer more fresh ideas in such networks if substantial qualitative differences are found in these subnetworks. Finally, the dynamics of individual career trajectories need to be considered in more depth using the overall approach we provide in this paper [39].

## Supporting information

S1 Fig Correlation matrix of the variables. (TIFF)

S2 Fig Histogram of our Gatekeeping variable. (TIFF)

S3 Fig The myopic nature of the Gatekeeping indicator. (TIFF)

S4 Fig Description of different moving-window settings. (TIFF)

S1 Table Descriptive statistics of our continuous variables. (TIFF)

S2 Table VIF-values in our final continuous model. (TIFF)

S3 Table Robustness check for k-core-based coreness measure. (TIFF)

S4 Table Robustness check for constraint-based Brokerage variable and the influence of Degree. (TIFF)

S5 Table Robustness check for only individual awards as dependent variable. (TIFF)

S6 Table Robustness check with randomly rewired networks and random award winners. (TIFF)

S7 Table Robustness checks based on rare event logit regression models. (TIFF)

S8 Table Robustness checks with individual level fixed effect models. (TIFF)

S9 Table Robustness check based on different moving windows. (TIFF)

S10 Table Robustness check with variables cut-off at 0.75 percentile. (TIFF)
