# Supplementary figures and images for: Brokering the core and the periphery: Creative success and collaboration networks in the film industry
Source: PLoS One. 2020 Feb 27;15(2):e0229436. doi: 10.1371/journal.pone.0229436 (PMC7046270; doi:10.1371/journal.pone.0229436)

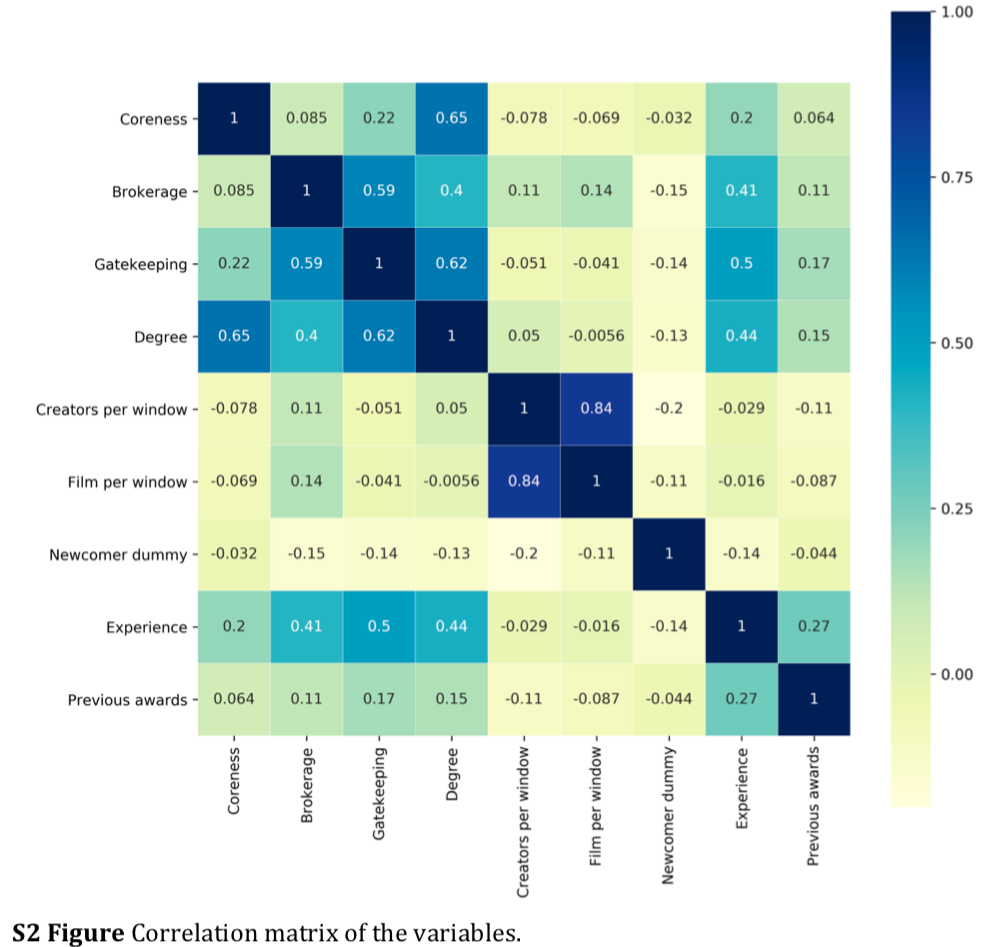

Supplement: S1 Fig — (TIFF) [file pone.0229436.s001.tiff]

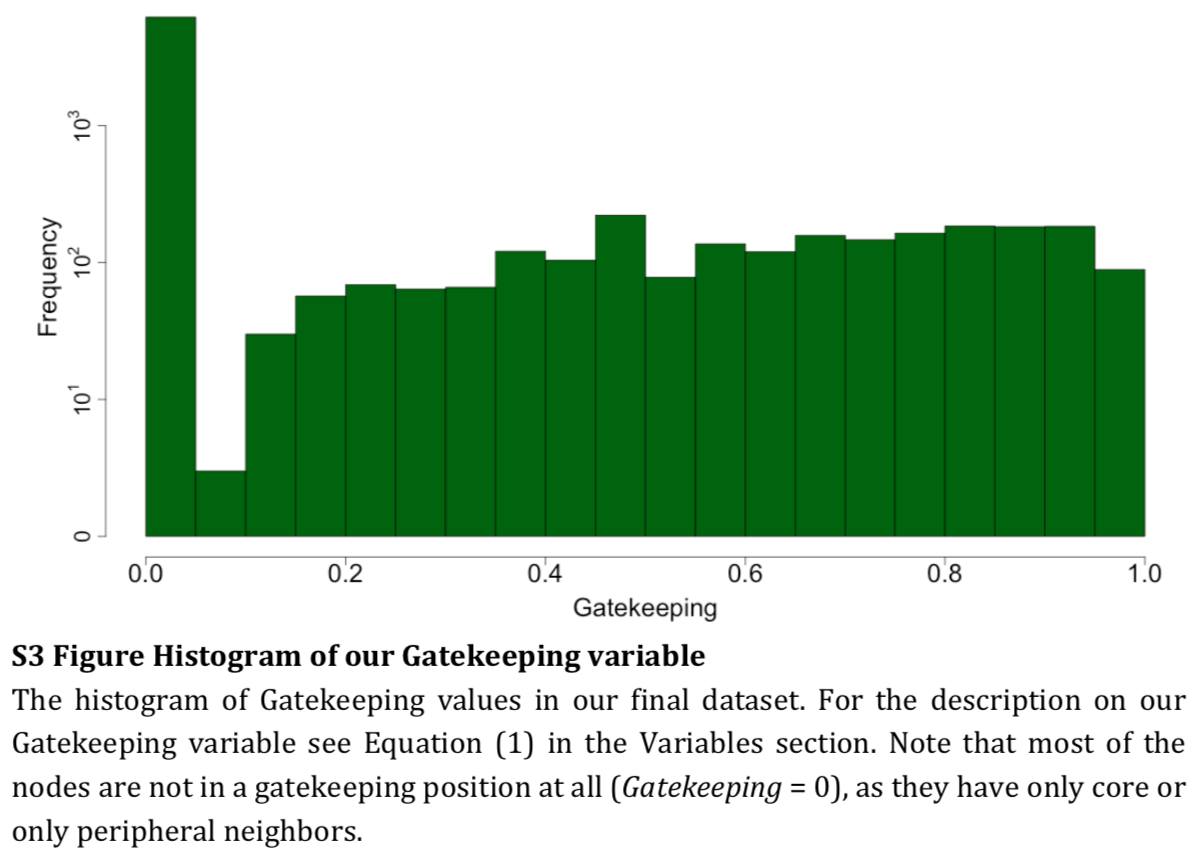

Supplement: S2 Fig — (TIFF) [file pone.0229436.s002.tiff]

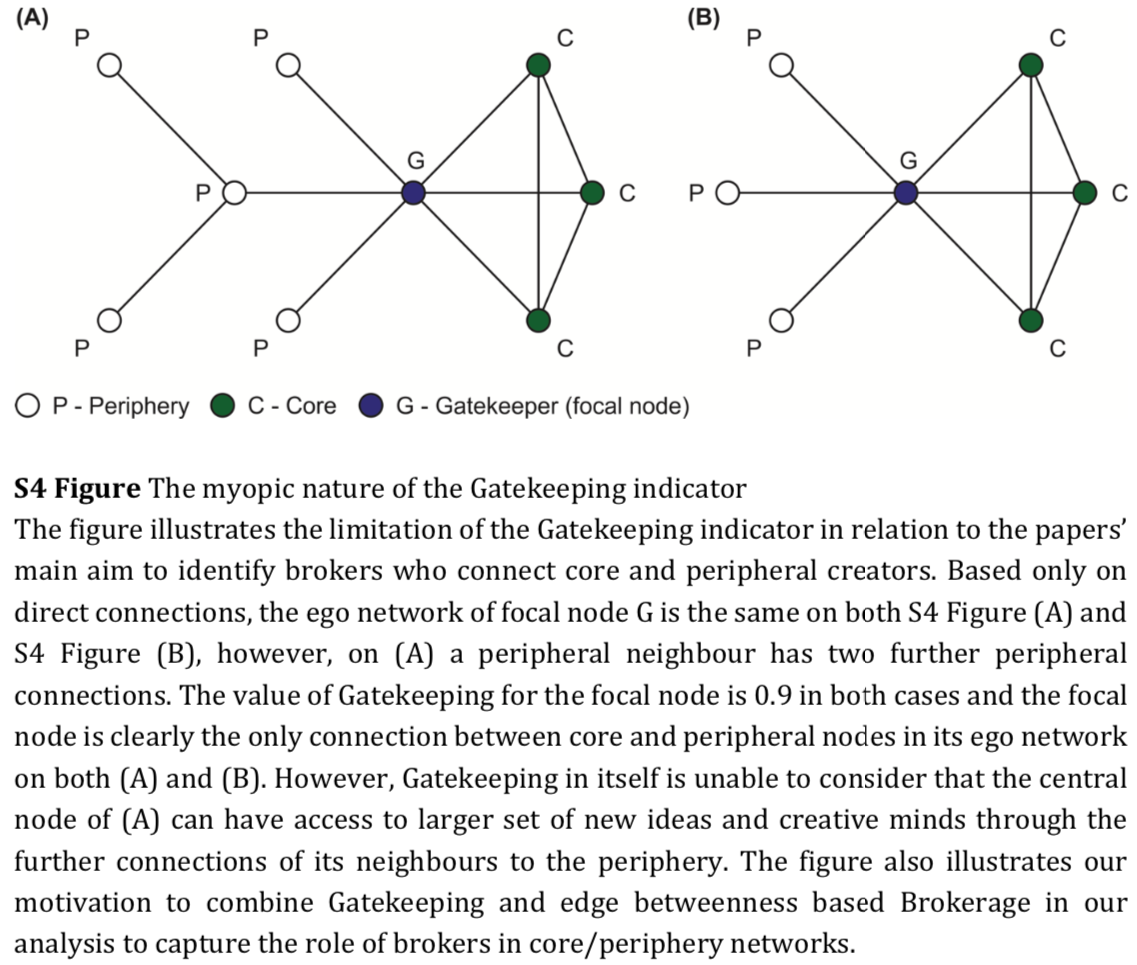

Supplement: S3 Fig — (TIFF) [file pone.0229436.s003.tiff]

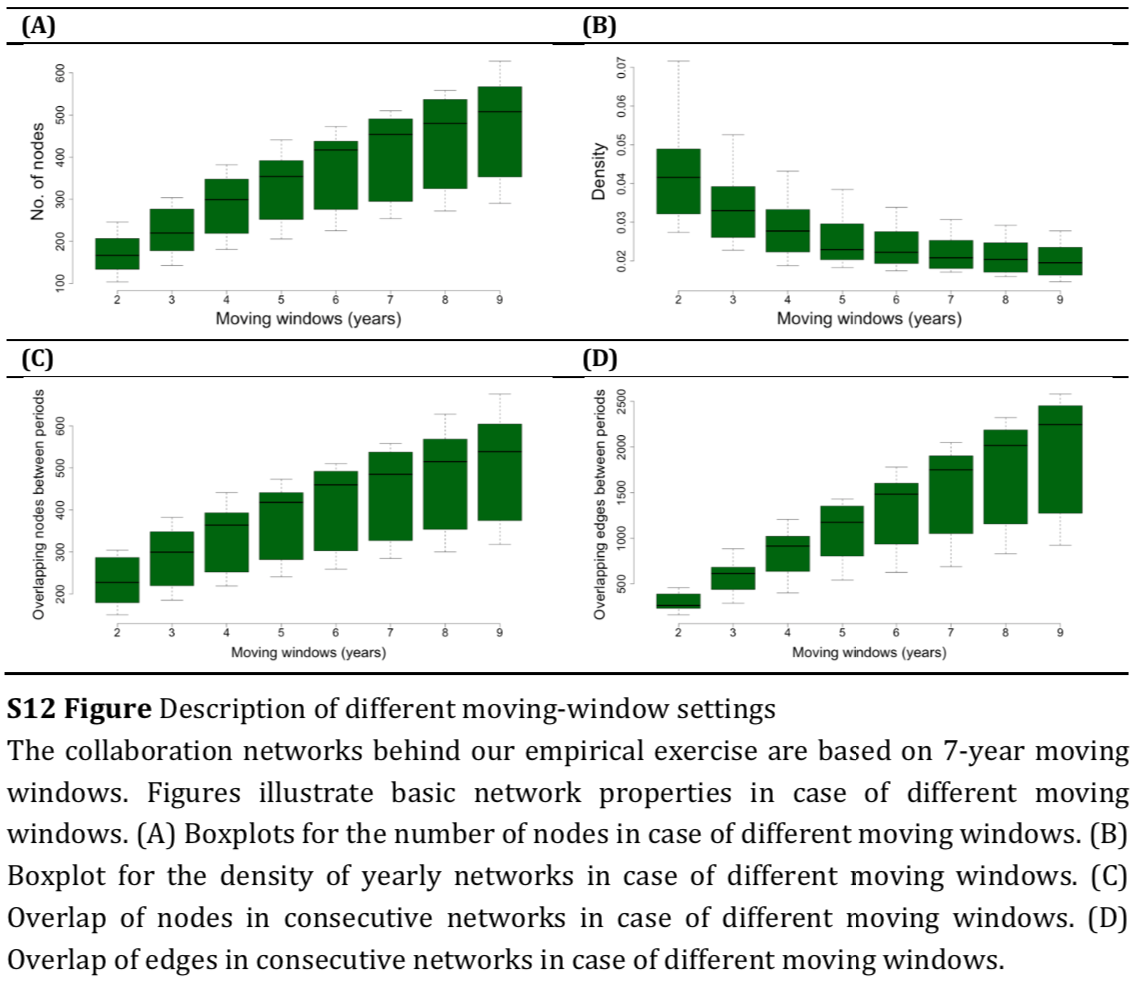

Supplement: S4 Fig — (TIFF) [file pone.0229436.s004.tiff]

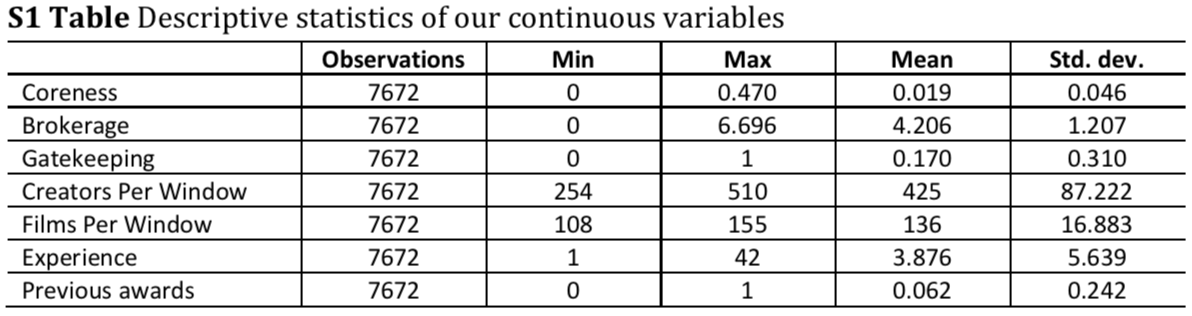

Supplement: S1 Table — (TIFF) [file pone.0229436.s005.tiff]

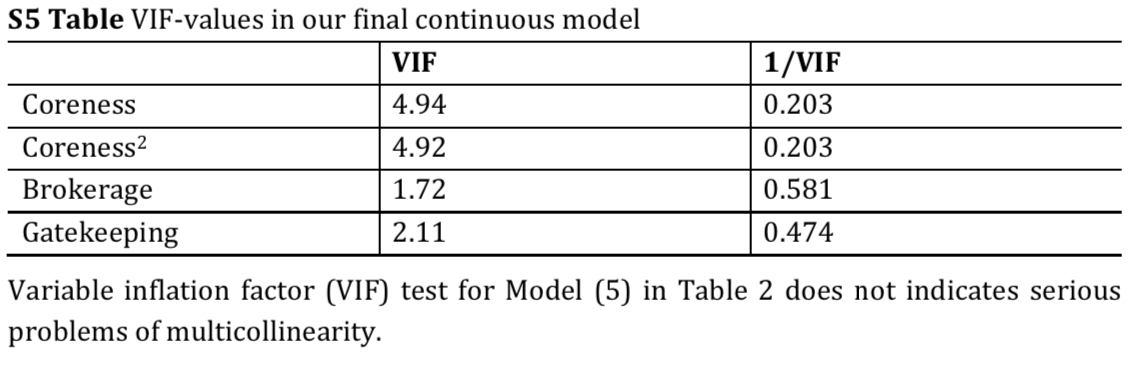

Supplement: S2 Table — (TIFF) [file pone.0229436.s006.tiff]

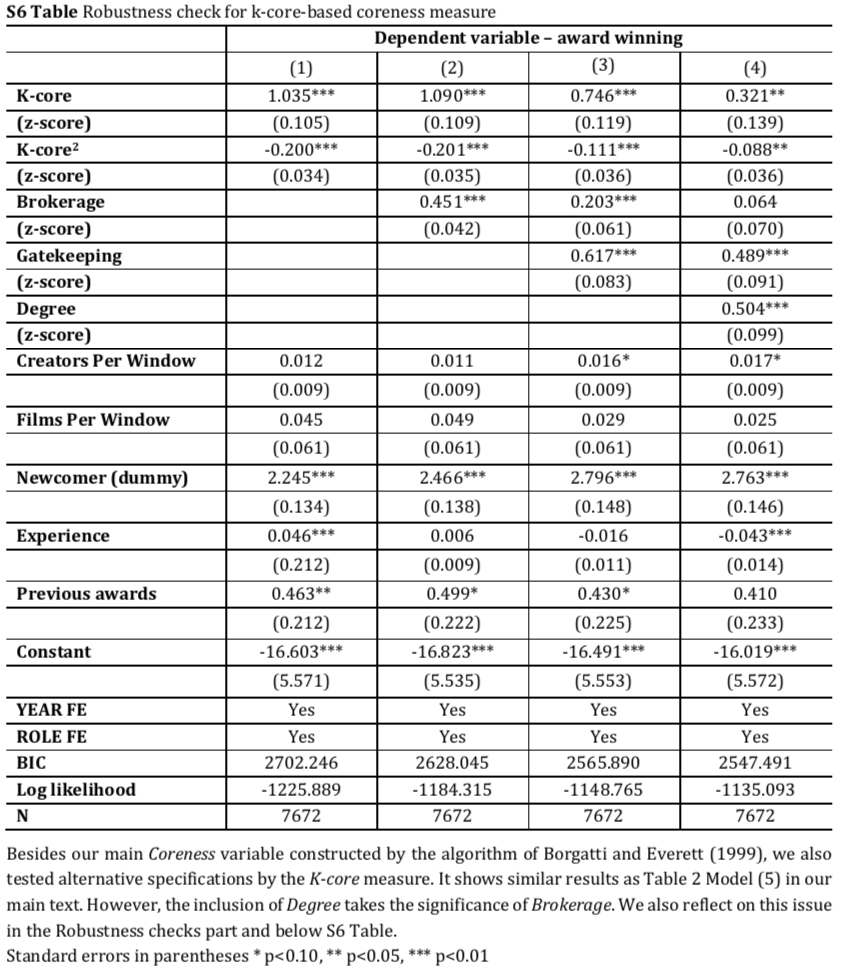

Supplement: S3 Table — (TIFF) [file pone.0229436.s007.tiff]

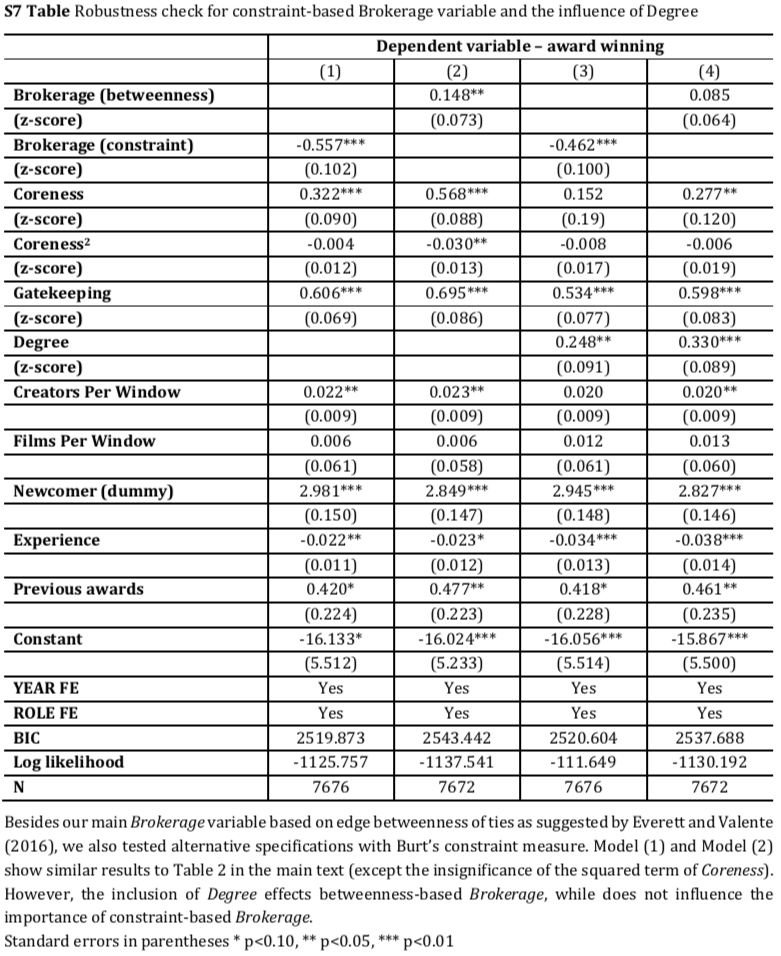

Supplement: S4 Table — (TIFF) [file pone.0229436.s008.tiff]

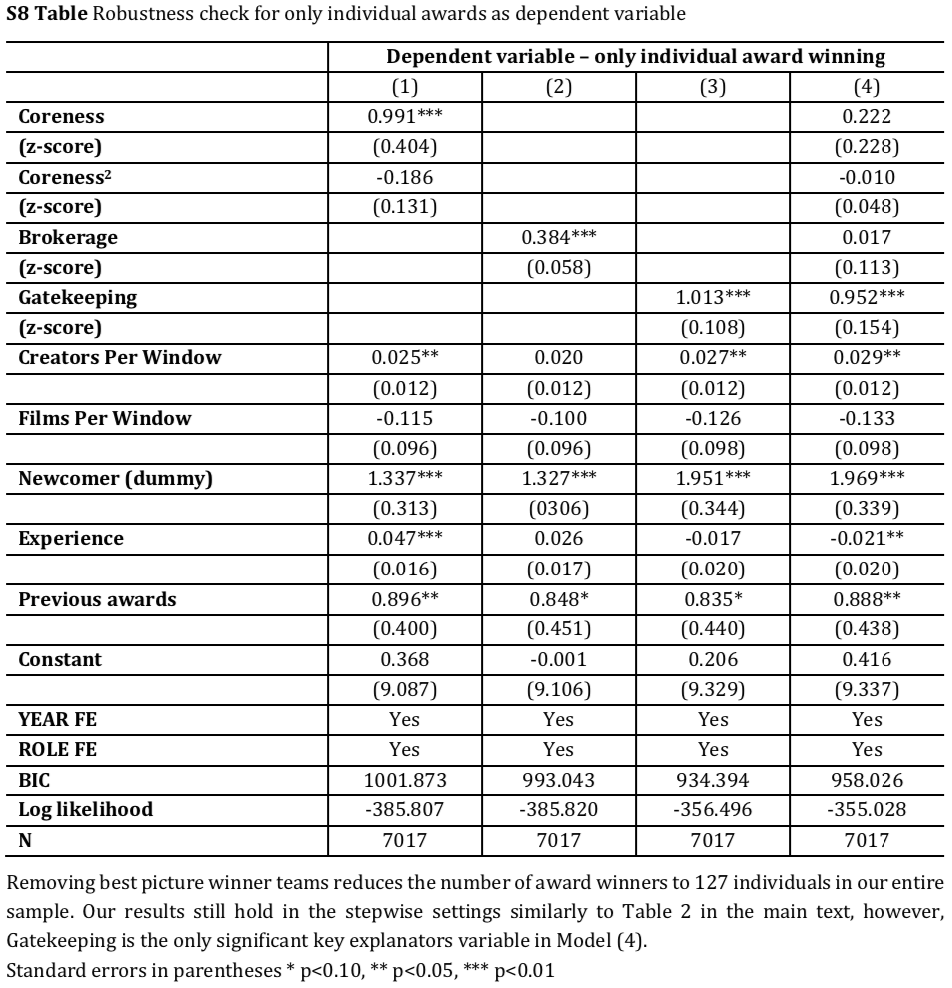

Supplement: S5 Table — (TIFF) [file pone.0229436.s009.tiff]

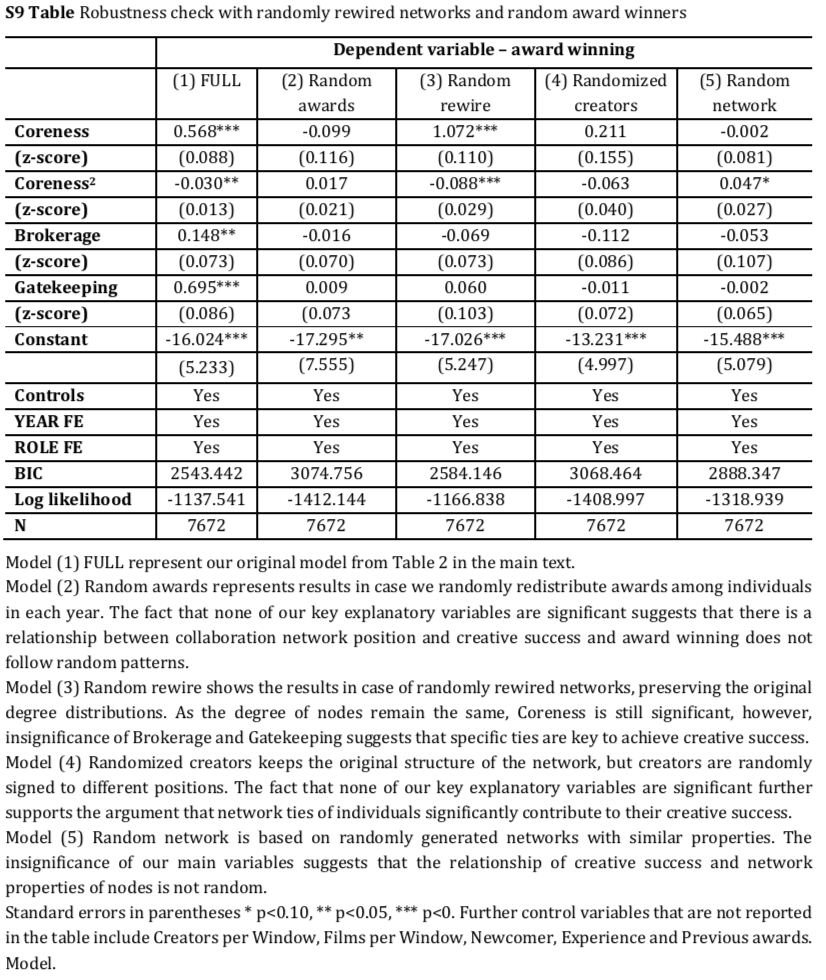

Supplement: S6 Table — (TIFF) [file pone.0229436.s010.tiff]

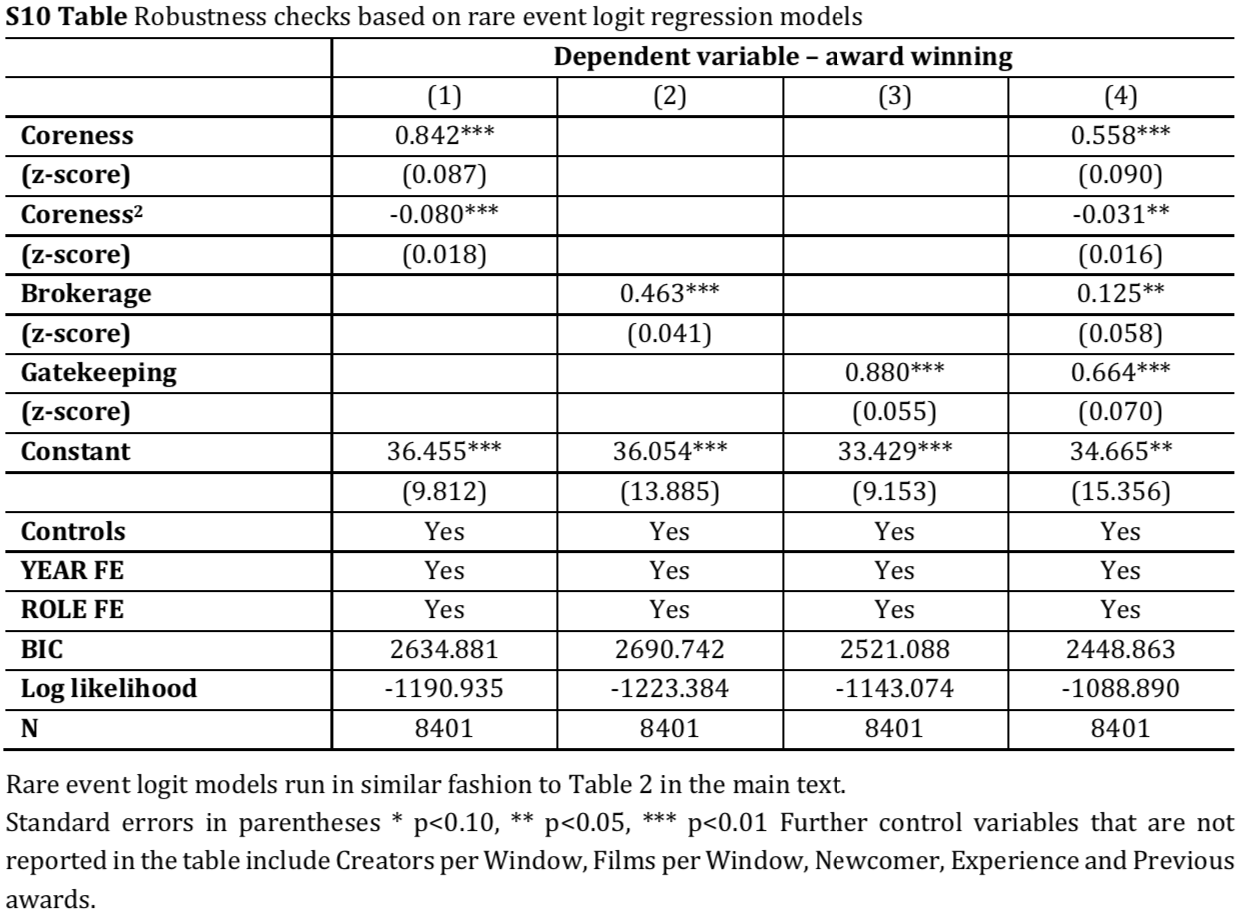

Supplement: S7 Table — (TIFF) [file pone.0229436.s011.tiff]

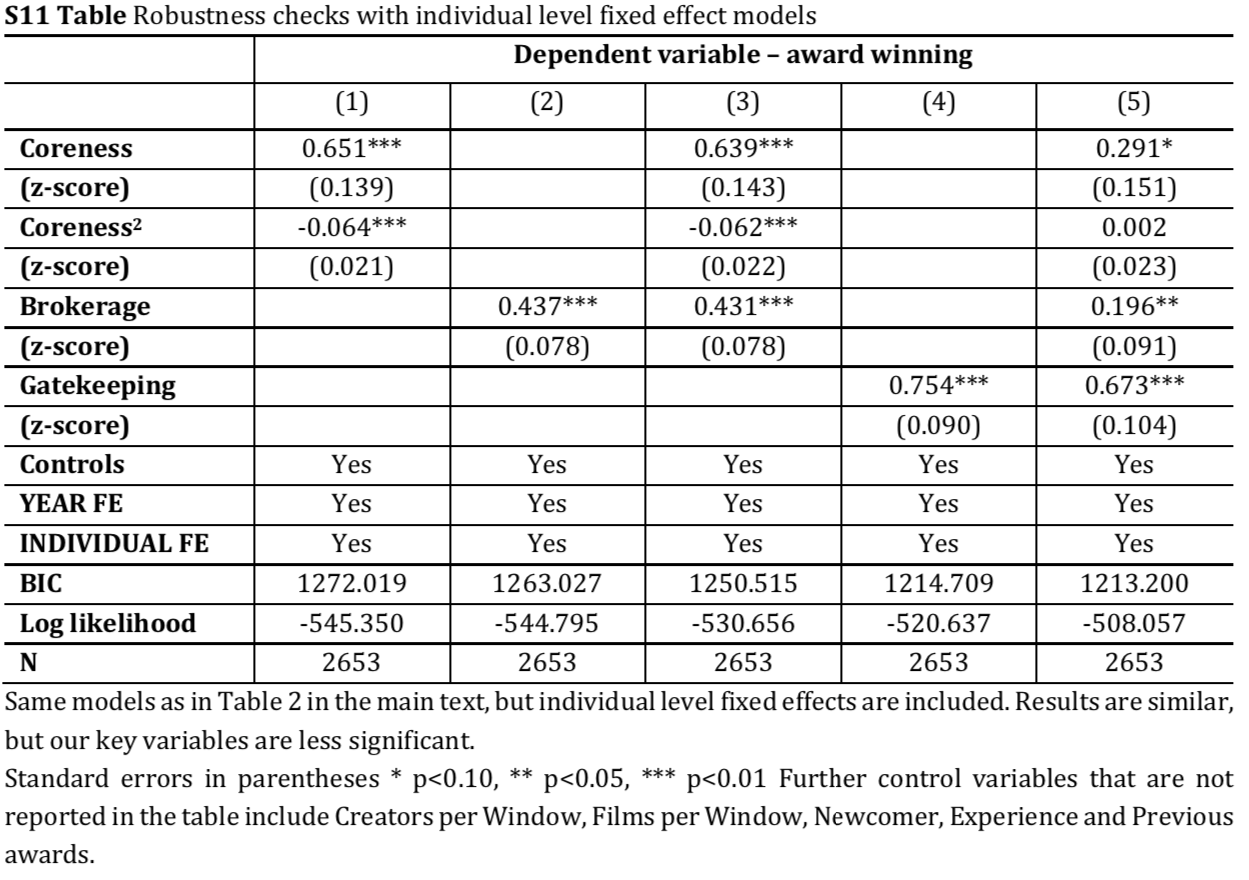

Supplement: S8 Table — (TIFF) [file pone.0229436.s012.tiff]

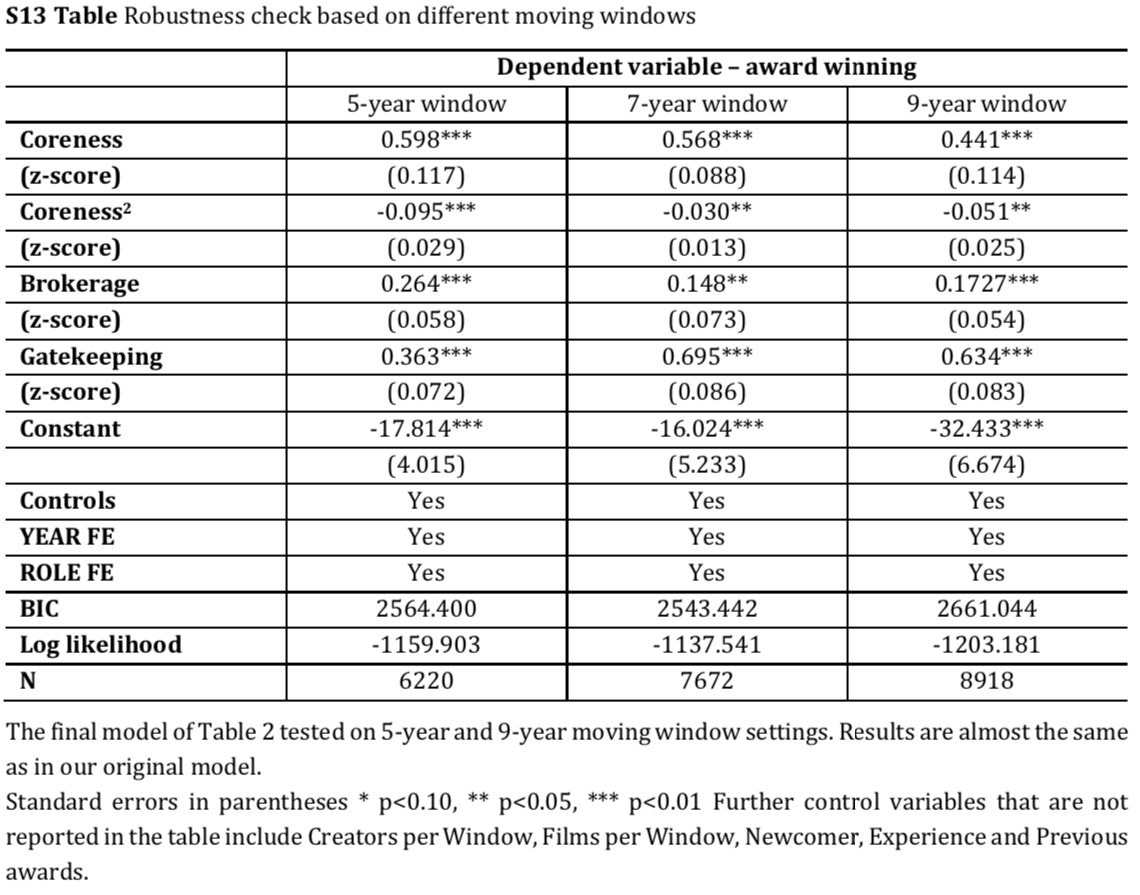

Supplement: S9 Table — (TIFF) [file pone.0229436.s013.tiff]

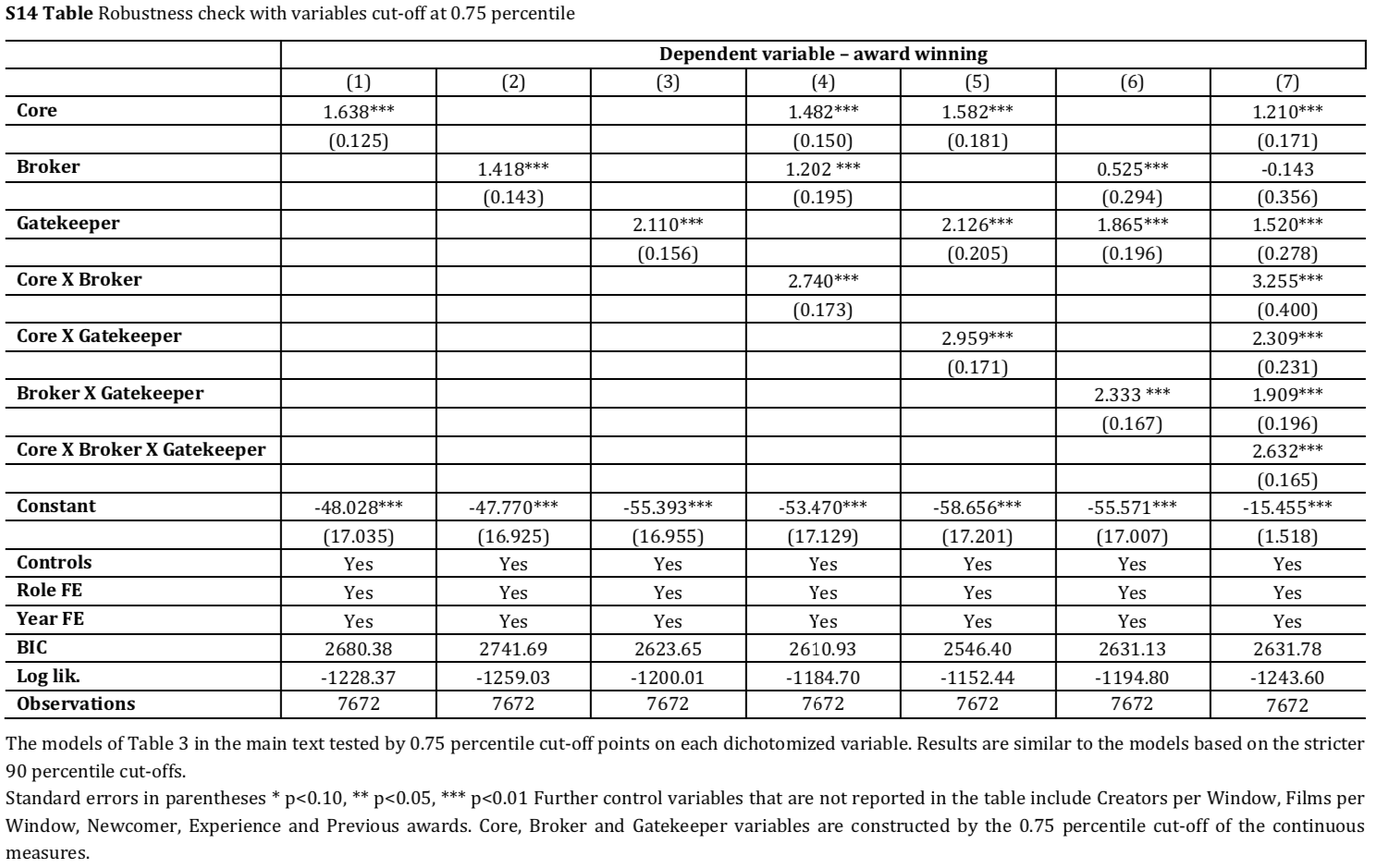

Supplement: S10 Table — (TIFF) [file pone.0229436.s014.tiff]
